# The Role of Context in Language Development for Children With Autism Spectrum Disorder

**DOI:** 10.3389/fpsyg.2020.563925

**Published:** 2020-12-23

**Authors:** Patricia Sánchez Pérez, Anders Nordahl-Hansen, Anett Kaale

**Affiliations:** ^1^Department of Special Needs Education, Faculty of Educational Sciences, University of Oslo, Oslo, Norway; ^2^Faculty of Education, Østfold University College, Halden, Norway; ^3^Norwegian Centre of Expertise for Neurodevelopmental Disorders and Hypersomnias, Oslo University Hospital, Oslo, Norway

**Keywords:** autism spectrum disorder, expressive language, context, home, preschool

## Abstract

Parent and preschool teacher ratings of the 10 noun categories of MacArthur-Bates Communication Development Inventory (CDI) were used to study expressive language in 2–4-year-old children with autism spectrum disorder (ASD) (*N* = 58) across the home and preschool context. There was no significant difference in the total number of words the children said in the two contexts, but the children said significantly more words in the noun categories “Furniture and rooms” and “People” at home. Only one third of the words the children said were said both at home and in the preschool, while the other two thirds were said only at home or only in preschool. This suggests that what words the children use across contexts differ substantially and that their vocabulary is larger than it seems when measured only in one context. This novel study highlights the importance of assessing the language in children with ASD in multiple contexts in order to better measure their vocabulary and to design appropriate language interventions.

## Introduction

There is growing evidence that development of expressive language in children with autism spectrum disorder (ASD) follows a qualitatively similar, but delayed pattern compared to children with typical development (TD) (Charman et al., 2003; Luyster et al., 2007; Weismer et al., 2010). Still, little is known about factors influencing early language in children with ASD. In typical development, variables such as the socioeconomic status of the main caregiver (e.g., Hoff, 2003; Rowe, 2012; Schwab and Lew-Williams, 2016) influence the early language of the child. Also, caregiver’s child-directed speech (e.g., Huttenlocher et al., 2010) and their diverse use of semantic categories (Jones and Rowland, 2017) show a strong impact on early language development. Tamis-LeMonda et al. (2019) observed how objects associated with what the child was doing during different home routines determined the semantic content of the child-directed speech of the mothers. They found that mothers were more likely to use words for toys during play with their child, words for food and utensils while feeding them, and words for body parts while grooming them. Even though decontextualized language emerges in typically developing children around 2 years of age (Uccelli et al., 2019), this might be delayed in children with ASD because these children often show delays in their general language development (e.g., Tager-Flusberg et al., 2005) and also have difficulties in generalizing from one context to another (Plaisted, 2015).

Most children with ASD attend preschool, and they will encounter many similar, but also some different objects, persons, and places across the home and preschool context. This object variation will influence what words parents and teachers use during their interaction with the child, which may subsequently affect the words used by young children with ASD. The aim of this study was to examine how expressive language of children with ASD might be different in various contexts. First, we compared the amount of words said by children with ASD at home and in preschool across 10 categories listing various types of objects, places, animals and persons that we assumed to be typical for one or the other context. Second, we investigated the degree of overlap between the words reported to be used by the children at home and in preschool. The results of this research may provide valuable insight into the role of context in the development of expressive language in young children with ASD.

## Method

### Participants

Fifty-eight 2–4 year old children (M = 48.8 months, SD = 8), 47 (81%) boys, with a confirmed ICD-10 diagnosis of childhood autism and their parents and preschool teachers participated in this study (Table 1). Children’s cognitive and language skills were assessed with the Mullen Scales of Early Learning (MSEL, Mullen, 1995) and the Norwegian translation of Reynell Developmental Language Scales (RDLS, Hagtvet and Lillestøen, 1985). The children had a mean mental age of 27.9 months (SD = 11.4), a mean receptive language age of 23.2 months (SD = 11.1), and a mean expressive language age of 21.1 months (SD = 11.9). Demographic data and information about the preschools was collected with questionnaires to parents and preschool teachers. Almost half of the mothers (26/46%) and fathers (23/44%) had a higher education degree. Forty-one (72%) were exclusively Norwegian-speaking homes, five (9%)were non-Norwegian-speaking homes and 11 (19%) spoke Norwegian and another language at home. The majority of children were Caucasian (40/69%), while the others had other backgrounds (8/14% Asian, 5/9% other/mixed, and 3/5% African). Most of the children (51/88%) attended a public mainstream preschool, while three (5%) attended a public preschool for children with ASD and four (7%) attended a unit for children with ASD in a public mainstream preschool. Their mean attendance in preschool was 37.3 h per week (SD = 5.1). The data used in the present study was a subset of the data collected for the baseline assessment in a previous study (Kaale et al., 2012). The Norwegian National Committee for Research Ethics approved the study. Participants provided written informed consent.

**TABLE 1:** Sample characteristics.

	**Mean/No. (%)**	**SD**	**Range**
Children			
Chronological age, overall	48, 8	8, 0	30–60
Mental age^1,2^, overall	27, 9	11, 4	9–59
2-year-olds (*n* = 6)	14, 2	4, 4	10–21
3-year-olds (*n* = 14)	26, 1	6, 7	18–43
4-year-olds (*n* = 37)	29, 5	12, 2	9–59
Receptive language age^3^, overall	23, 2	11, 1	6–60
2-year-olds (*n* = 6)	11, 2	7, 3	9–24
3-year-olds (*n* = 14)	22, 8	7, 7	9–36
4-year-olds (*n* = 38)	24, 2	11, 9	6–60
Expressive language age^3^, overall	21, 1	11, 9	3–60
2-year-olds (*n* = 6)	12, 6	6, 5	8–24
3-year-olds (*n* = 14)	19, 4	5, 8	10–30
4-year-olds (*n* = 38)	22, 3	13, 1	3–60
Gender			
Female	11 (19%)		
Male	47 (81%)		
Hours in preschool per week^4^	37, 3	5, 1	20–45
Parents			
Mother’s educational level^5^			
Primary education	8 (14%)		
Secondary education	22 (40%)		
University/College	26 (46%)		
Father’s educational level^6^			
Primary education	5 (9%)		
Secondary education	24 (45%)		
University/College	23 (44%)		
Language spoken at home^7^			
Norwegian only	41 (72%)		
Norwegian and another	11 (19%)		
Other than Norwegian	5 (9%)		

*^1^Mullen Scale of Early Learning (MSLE).^2^Missing data from one child.^3^Reynell Developmental Language Scale (RDLS); for scores <4 stanine for 1.5 years language age was based on MSLE.^4^Missing data from two children.^5^Missing data from two mothers.^6^Missing data from six fathers.^7^Missing data from five fathers and one mother.*

### Measures

The Communicative Development Inventories “Words and Gestures” form (CDI-WG; Fenson et al., 1994), completed by parents and preschool teachers, were used to measure the words the children said at home and in the preschool. The CDI forms were sent separately to parents and preschool teachers, along with instructions on how to fill them in, and they were collected upon arrival the day of cognitive and language assessments. CDI includes a checklist with 396 vocabulary items across 19 different semantic categories including nouns, verbs, adjectives, pronouns, prepositions and quantifiers. In the present study, only the 10 categories containing exclusively nouns were used (e.g., “Toys,” “Clothes,” “Rooms and Furniture,” and “Small things in the household”). Objects, persons and places listed in most of the categories are equally typical for home and preschool, but some of the noun categories are more typical for one context than the other. Words from the categories “People” (e.g., aunt and babysitter), “Rooms and furniture” (e.g., bedroom and living room) and “Small things in the household” (e.g., towel, vacuum cleaner) are more typical for the home context. The amount of words varied from eight to 36 in the noun categories, and across the 10 noun categories there were a total of 228 words. Based on the CDI data from parents and preschool teachers we calculated the number of words said by the child at home and in the preschool across the 10 noun categories and for each category. We also calculated “matching” variables defined as the number of words the children said both at home *and* in the preschool in each of the 10 noun categories. In addition, we calculated the number of “unique words” the children said across the two contexts. This was computed based on the number of words reported by parents plus the number of words reported by preschool teachers minus the number of words reported by both of them (the “matching”). Last, we computed the percentage for the “matching” variables (i.e., number of words reported by parents plus preschool teachers divided by the number of “unique words” multiplied by 100). The CDI has previously shown high concurrent validity with direct assessments (Nordahl-Hansen et al., 2014) and high inter-rater reliability (Nordahl-Hansen et al., 2013) when used with children with ASD.

### Statistical Analyses

Along with descriptive statistics, paired sample *t*-tests were performed to compare the differences in the number of words the children were reported to say at home and in the preschool both overall and across the 10 noun categories. In addition, two paired sample *t*-tests were conducted to compare the number of words reported to be said at home and in preschool combined with the number of words reported to be said only by parents and only by preschool teachers, respectively. We also ran descriptive analyses on the “matching” variable (i.e., mean percentages). The software IBM SPSS version 25 and Microsoft Excel 2016 were used to analyze the data.

## Results

There was no significant difference in the overall number of words the children said at home and in the preschool (M_*home*_ = 78.1, SD = 78.4; M_*preschool*_ = 70.5, SD = 75.9, and *p* = 0.07) (Table 2). The same was true for most of the 10 noun categories, except for “Furniture and rooms” (M_*home*_ = 7.4, SD = 8.8; M_*preschool*_ = 5.4, SD = 7.4, and *p* = 0.00) and “People” (M_*home*_ = 6.0, SD = 5.5; M_*preschool*_ = 5.0, SD = 5.8, and *p* = 0.04), which were significantly different. Parents and preschool teachers reported that the children said one third of the 228 words listed in the 10 noun categories at home (M_*home*_ = 34%, SD = 34%) and in the preschool (M_*preschool*_ = 31%, SD = 33%). The highest percentages of listed words reported to be said by both parents and preschool teachers were in the noun categories “Vehicles (real or toy),” “Food and drinks,” and “Toys.”

**TABLE 2:** Number of words parents and preschool teachers report that the children (*N* = 58) say at home and in preschool across all 10 noun categories and for each category.

**Children Development Inventory (CDI) categories**	**Home mean (SD)/%^2^**	**Preschool mean (SD)/%**	**Mean diff. (SD)/%**	***t*(df), *p***
Across all the 10 noun categories (228)^1^	78.1 (78.4)/34	70.5 (75.9)/31	7.6 (31.5)/3	1.8 (57), *p* = 0.07
Animals (real or toy) (36)	11.6 (12.3)/32	11.5 (12.3)/32	0.1 (6.4)/0	0.3 (57), *p* = 0.87
Vehicles (real or toy) (9)	4.4 (3.6)/49	4.1 (3.8)/46	0.3 (1.6)/3	1.5 (57), *p* = 0.15
Toys (8)	3.2 (3.2)/40	3.3 (3.2)/41	−0.1 (1.4)/−1	−0.5 (57), *p* = 0.65
Food and drinks (30)	12.2 (11.4)/41	11.1 (10.8)/37	1.1 (4.6)/4	1.8 (57), *p* = 0.09
Clothes (19)	6.3 (6.9)/33	6.0 (6.8)/31	0.3 (3.5)/2	0.6 (57), *p* = 0.53
Body parts (20)	7.7 (8.0)/38	7.2 (8.1)/36	0.4 (3.9)/2	0.9 (57), *p* = 0.40
Furniture and rooms (24)	7.4 (8.8)/31	5.4 (7.4)/23	2.0 (4.7)/8	**3.2 (57), *p* = 0.00**
Small things in the household (36)	11.4 (12.7)/32	9.7 (12.0)/27	1.6 (6.3)/5	2.0 (57), *p* = 0.06
Outside things and places to go (26)	8.1 (9.0)/30	7.2 (8.7)/27	0.9 (4.4)/3	1.5 (57), *p* = 0.15
People (20)	6.0 (5.5)/30	5.0 (5.8)/25	1.0 (3.8)/5	**2.1 (57), *p* = 0.04**

*^1^Number in brackets in this column indicates total amount of words in that semantic category.^2^Percentage of total possible words the parents/preschool teachers report that the child say (i.e., number of words reported × 100/total number of words in the category). The values in bold represent statistical significance.*

**FIGURE 1:**
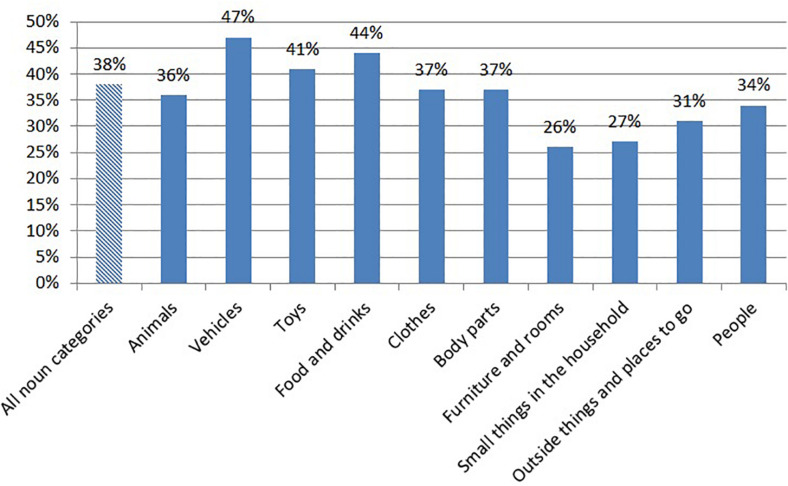
Percentages of words said both at home and in the preschool (match) across all 10 noun categories and for each category.

After analyzing the “matching” variables, we found that among the words parents and preschool teachers reported that the children said, 38% was said both at home and in the preschool (ranging from 26 to 47% across the 10 categories/Figure 1). The match was lowest for “Furniture and rooms” (26%) and “Small things in the household” (27%), and highest for “Vehicles (real or toy)” (47%) and “Food and drinks” (44%). For example, the children were reported to say on average 12.2 and 11.1 words in the category “Food and drinks” at home and in the preschool, respectively, but only 44% (5.2) of these words were reported to be said in both contexts. This suggests that the children said 7.0 “Food and drinks” words at home that they did not say in the preschool and 5.9 words in preschool that they did not say at home. This means that the actual number of “Food and drinks” words the children said was closer to 18 (5.2 words said both at home and in preschool + 7.0 words said only at home + 5.9 words said only in preschool = 18.1 words). This pattern was true for all 10 noun categories. In fact, the children said a total of 91, 7 words (SD = 86, 8) if adding together words said in both contexts, words said only at home and words said only in preschool. There was a significant difference between the total number of words reported at home and preschool combined and both the number of words reported only by parents [M_*difference*_ = 13.5, SD = 16.5, *t*(57) = 6.23, and *p* < 0.000] and the number of words reported only by preschool teachers [M_*difference*_ = 21.1, SD = 27.4, *t*(57) = 5.89, and *p* < 0.000]. Thus, the children’s vocabulary was larger than what was captured by relying on reports only from parents or preschool teachers.

## Discussion

This study aimed to investigate how expressive language of young children with ASD might be different in various contexts using CDI completed by parent and preschool teachers. We found that the overall number of words said at home and in the preschool was quite similar. Both parents and preschool teachers reported that the children said most words in the categories “Toys,” “Vehicles (real or toy),” and “Food and drinks.” This could be explained by the fact that play and food are two important areas in the first years of life, and objects, and therefore words related to these areas, are probably of high frequency in the learning contexts of these children. We also found that the children used more words from the “Furniture and Rooms” and “People” categories at home compared to preschool. This finding is in line with our expectations as we assumed that objects belonging to these two categories are more prevalent at home, and thus will affect the caregiver’s child-directed speech, which next supports the children’s use of these words (Huttenlocher et al., 2010). Interestingly, we found that only one third of the words the children said were used both at home and in the preschool and two thirds only in one or the other context. This suggests that language during the first 4 years of life in children with ASD is context-dependent, which is similar to what is found in younger children with typical development (Uccelli et al., 2019). This interpretation is further supported by the fact that the highest proportions of words said across both contexts were in the categories “Toys,” “Food and Drinks,” and “Vehicles (real or toy),” which are essential objects both at home and in the preschool. This supports Hills et al. (2009) notion of “preferential acquisition” as the working principle behind language development: words that are better interconnected in the learning context, rather than in the child’s internal semantic network, are learned earlier in development. When adding together all the words the children were reported to say both at home and in preschool, only at home and only in preschool, we found that the children vocabulary was significantly larger compared to measuring their vocabulary only at home or only in preschool. This suggests that the vocabulary of young children with ASD may be larger than what is revealed by investigating only one context, which is currently the most common way to collect information about everyday language of children with ASF. In a previous study, we reported high interrater reliability between parent and preschool teacher reports (Nordahl-Hansen et al., 2013), but the reliability was then calculated using total amount of words reported by parents and teachers, not the actual words the children say across the home and preschool contexts. The finding of the present study suggests that a cumulative CDI score from multiple sources such as parents and preschool teachers combined will give a better picture of children’s language abilities.

### Limitations

The expressive language of children with ASD has previously been studied with the CDI (e.g., Charman et al., 2003), but this is the first study to use this instrument to measure expressive language across contexts by comparing data from two sources for each child. Still, the study has some limitations. First, we did not include a comparison group of children with typical development or children with developmental language disorder. Therefore we do not know if the findings are unique to children with ASD, although based on previous research (Charman et al., 2003; Luyster et al., 2007; Luyster and Arunachalam, 2018) we expect that the same pattern will be evident in younger children with TD. Second, it might be that parents and preschool teachers are more prone to report that children say words related to objects in their environment, and that the findings do not reflect the words the children say, but a recall bias in parents and teachers. A more narrow age range among the study participants (e.g., only 4-year-olds versus 2–4-year-olds as in the present study) might have helped making the results more precise. However, as children with ASD are known to be very heterogeneous regarding their language development recruiting only 4-year-olds would pose some of the same challenges compared to TD samples. Also, although the CDI has shown high concurrent validity compared to both direct structured tests and language samples (Dale, 1991; Nordahl-Hansen et al., 2014), observational data of factual word use in both contexts would have strengthened the findings. Last, the findings are based on parents and teachers reports of the words the children produce only at one time point so we do not know how stable the results are.

### Future Directions

Future research should include a comparison group of children with typical development who are matched both on chronological and mental age. Other interesting aspects would be to investigate which specific words within the 10 categories are typically used in one of these two contexts but not in the other (there might be even a pattern), the influence of the language spoken at home and the use of words across the two contexts in subgroups of children with ASD (e.g., those who are just starting to speak and those with a more developed vocabulary).

## Data Availability Statement

The data analyzed in this study is subject to the following licenses/restrictions: Not available due to restrictions related to ethical regulations. Requests to access these datasets should be directed to AK, anett.kaale@isp.uio.no.

## Ethics Statement

This study was reviewed and approved by the Norwegian National Committee for Research Ethics. Written informed consent was provided by parents and preschool teachers.

## Author Contributions

The data used in this study was originally gathered for a RCT lead by AK. All authors contributed in writing the manuscript and took part in the design and analyses of this study.

## Conflict of Interest

The authors declare that the research was conducted in the absence of any commercial or financial relationships that could be construed as a potential conflict of interest.
